# Causal Inference and Annotation of Phosphoproteomics Data in Multiomics Cancer Studies

**DOI:** 10.1016/j.mcpro.2025.100905

**Published:** 2025-01-09

**Authors:** Qun Dong, Minjia Tan, Yingchun Zhou, Yue Zhang, Jing Li

**Affiliations:** 1 Department of Bioinformatics and Biostatistics, School of Life Sciences and Biotechnology, Shanghai Jiao Tong University, Shanghai, China; 2 State Key Laboratory of Drug Research, Shanghai Institute of Materia Medica, Chinese Academy of Sciences, Shanghai, China; 3 University of Chinese Academy of Sciences, Beijing, China; 4 Zhongshan Institute for Drug Discovery, Shanghai Institute of Materia Medica, Chinese Academy of Sciences, Guangdong, China; 5 Key Laboratory of Advanced Theory and Application in Statistics and Data Science - MOE, School of Statistics, East China Normal University, Shanghai, China

**Keywords:** phosphoproteomics, multiomics, causal inference, network, cancer proteomics

## Abstract

Protein phosphorylation plays a crucial role in regulating diverse biological processes. Perturbations in protein phosphorylation are closely associated with downstream pathway dysfunctions, whereas alterations in protein expression could serve as sensitive indicators of pathological status. However, there are currently few methods that can accurately identify the regulatory links between protein phosphorylation and expression, given issues like reverse causation and confounders. Here, we present Phoslink, a causal inference model to infer causal effects between protein phosphorylation and expression, integrating prior evidence and multiomics data. We demonstrated the feasibility and advantages of our method under various simulation scenarios. Phoslink exhibited more robust estimates and lower false discovery rate than commonly used Pearson and Spearman correlations, with better performance than canonical instrumental variable selection methods for Mendelian randomization. Applying this approach, we identified 345 causal links involving 109 phosphosites and 310 proteins in 79 lung adenocarcinoma (LUAD) samples. Based on these links, we constructed a causal regulatory network and identified 26 key regulatory phosphosites as regulators strongly associated with LUAD. Notably, 16 of these regulators were exclusively identified through phosphosite–protein causal regulatory relationships, highlighting the significance of causal inference. We explored potentially druggable phosphoproteins and provided critical clues for drug repurposing in LUAD. We also identified significant mediation between protein phosphorylation and LUAD through protein expression. In summary, our study introduces a new approach for causal inference in phosphoproteomics studies. Phoslink demonstrates its utility in potential drug target identification, thereby accelerating the clinical translation of cancer proteomics and phosphoproteomic data.

## Highlights

• Phoslink is a computational approach for causal inference in phosphoproteomics.
• Phoslink outperforms canonical Mendelian randomization and correlation methods.
• Phoslink uncovers novel regulatory phosphosites and their network.
• Phoslink is available as an R package.

## Graphical Abstract

**Figure:**
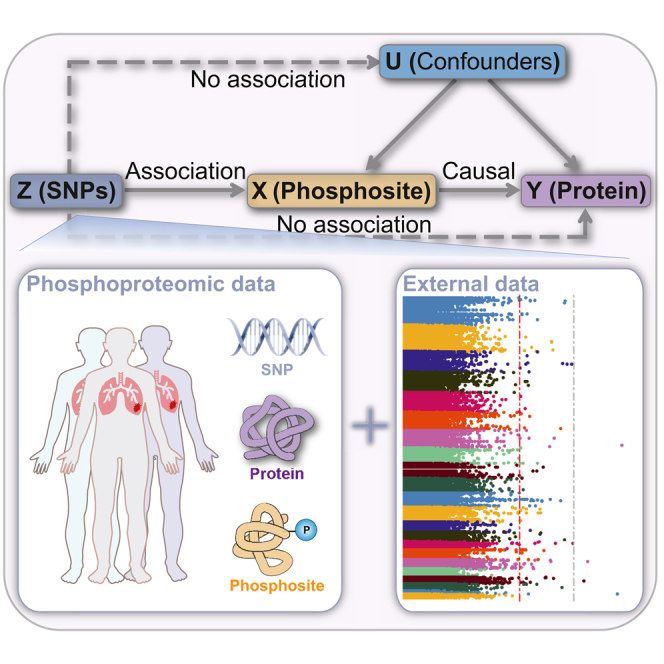

## In Brief

Despite mass spectrometry detecting numerous phosphosites, over 95% remain uncharacterized. Our study introduces Phoslink, which uses protein expression as a sensitive indicator of cellular states, identifying causally regulated phosphosites that affect protein expression with greater precision and robustness. Applied to CNHPP-LUAD, Phoslink uncovers novel regulatory phosphosites with critical implications for LUAD and drug repurposing. Phoslink is a powerful tool for causal inference in phosphoproteomics, offering new opportunities to advance our understanding of phosphosites' functions and accelerate clinical translation.

Phosphorylation is the most common post-translational modification, with estimates suggesting that over two-thirds of human proteins may be phosphorylated (1). This reversible modification as a dynamic molecular switch modulates diverse protein functions by inducing conformational changes and further activates or inactivates downstream signal transduction cascades. Protein phosphorylation is involved in regulating nearly all cell processes, and aberrant phosphorylation is intimately associated with multiple pathological conditions, particularly cancer (2). For example, Rb phosphorylation drives colon cancer proliferation and could serve as a promising therapeutic target (3).

Increasingly sensitive mass spectrometry (MS)–based technologies have enabled the detection of phosphosites at a large scale within a single cohort dataset, as seen in studies such as the Chinese Human Proteome Project (CNHPP) and Clinical Proteomic Tumor Analysis Consortium (CPTAC). However, the functions and potential disease risks of a majority of these phosphosites remain unknown, as less than 5% have been annotated with specific functions (4). An imbalance in protein phosphorylation can disrupt downstream biological pathways, and alterations in protein expression could serve as an indicator for the status of various cellular processes (5), such as cancerous transformation of cells. Therefore, it is critical to identify and understand the regulatory relationships between phosphorylation and protein expression in cancer research.

In cancer multiomics research, the relationships among biomolecules are typically characterized through correlation analysis such as Pearson and Spearman (6, 7). In our previous work, we conducted the large-scale proteomic landscape of lung adenocarcinoma (LUAD) in Chinese patients (CNHPP-LUAD cohort), which deepened the understanding of molecular characteristics and provided clues for novel diagnostic and therapeutic strategies. Nevertheless, the regulation of phosphorylation remains unresolved. Similar to previous studies in tumor proteomics, we established links between phosphorylation and protein expression based on Spearman correlation analysis (8). However, an observational correlation between phosphorylation level and protein expression does not necessarily imply a causal relationship, which bears major responsibility for the failure of certain kinase-targeted inhibitors to achieve desired outcomes in clinical trials (9). There are several studies currently investigating causal relationships among molecules, such as CausalPath (10) and CARNIVAL (11), which infer causal associations based on known pathways and changes in gene or protein expression profiles. Consequently, these methods remain incapable of inferring the novel connections that have not been reported in the prior pathways. Besides, establishing causality within observational data can be challenging because of issues like reverse causation and confounders. Mendelian randomization (MR) has emerged as a promising approach to obtaining valid causal inferences from observational data. MR is grounded in the Mendelian genetic law and utilizes germline genetic instrumental variables (IVs), usually SNPs, to assess causal effects in observational datasets. These SNPs are randomly assorted during gamete formation and conception, providing a naturally randomized comparison (Supplemental Fig. S1). Their fixed nature at conception prevents modification by subsequent factors (12, 13). Consequently, MR is robust against biases like reverse causation and confounders in observational studies, enabling valid causal inference from such data. While most MR investigations utilize summarized data from consortia to leverage their large sample sizes, such as genome-wide association studies (GWAS) (14), the application of MR in cancer research is limited by the relatively smaller sample sizes of multiomics cancer cohorts compared with GWAS (15). This limitation poses a challenge in identifying reliable IVs. Consequently, the development of an alternative IV selection method tailored for multiomics cancer studies is critically needed.

In this study, we present a causal inference model called “Phoslink” for inferring causal effects between protein phosphorylation and downstream protein expression. To select reliable IVs for MR in the small-sample cancer proteomics dataset, our approach incorporates the prior evidence from GWAS and PhosSNP (16). Extensive simulations with different causal effect sizes, sample sizes, and heterogeneity of data were conducted to evaluate the feasibility and performance of Phoslink. Phoslink was demonstrably superior in controlling the risk of false positives. Moreover, Phoslink could yield more robust estimates and lower false discovery rates (FDRs) than commonly used correlation-based methods. Phoslink could serve as an effective tool for causal inference of key phosphosites in human proteomics studies and facilitate drug target discovery.

## Experimental Procedures

### Simulation Study Design

To assess the performance and reliability of Phoslink, we conducted simulation studies using a series of scenarios with different parameter settings. For each individual *i*, let $X_{i}$ denote the exposure (represent phosphorylation of phosphosite in the real data analysis), $Y_{i}$ denote the outcome (represent protein expression as in the real data analysis), and $U_{i}$ represent a potential confounder of the exposure–outcome relationships. Let $G_{ij}$ be the genotype of ${\text{SNP}}_{j}$. Referring to most MR data–generating models (17, 18, 19), the simulations are conducted based on the following model:

$$U_{i}=\sum_{j=1}^{J}\Phi_{j}G_{ij}+\epsilon_{i}^{U}$$

$$X_{i}=\sum_{j=1}^{J}\gamma_{j}G_{ij}+{\theta_{Ux}U}_{i}+\epsilon_{i}^{X}$$

$$Y_{i}=\sum_{j=1}^{J}\alpha_{j}G_{ij}+\theta X_{i}+{\theta_{Uy}U}_{i}+\epsilon_{i}^{Y}$$

$G_{ij}$ ∼ Binomial(2, 0.3) independently

$\epsilon_{i}^{U}$, $\epsilon_{i}^{X}$, $\epsilon_{i}^{Y}$ ∼ *N*(0, 1) independently

where $\Phi_{j}$ represents the effect of the genetic variant on the confounder **U**, $\gamma_{j}$ represents the genetic effect of $G_{j}$ on **X**, $\alpha_{j}$ represents the direct effect of the genetic variant $G_{j}$ on the outcome **Y**, and *θ* is the causal effect of **X** on **Y**. The effects of the confounder **U** on **X** and **Y** are denoted by $\theta_{Ux}$ and $\theta_{Uy}$, respectively. The genetic effects of SNPs on the exposure were drawn from a uniform distribution *U*(0.08, 0.10). We set a total of 50 SNPs, among which 30 were designated as IVs. We randomly selected 0%, 30%, and 50% of the IVs to be invalid IVs. SNPs with direct effects on the outcome, violating the exclusion restriction assumption, are considered invalid IVs. The parameter $\alpha_{j}$ was generated from a normal distribution *N*(0, 0.15) and $\Phi_{j}$ = 0 for invalid IVs and the $\Phi_{j}$ = $\alpha_{j}$ = 0 for valid IVs. We fixed $\theta_{Ux}$ and $\theta_{Uy}$ at 0.75.

The simulation data comprised two parts. In the first part, we simulated a GWAS dataset by generating exposure **X** and SNP data for 100,000 samples. The second part involved simulating a multiomics cancer dataset (X′, SNP', and Y′). Considering population heterogeneity, which refers to the variability between the simulated GWAS and small-sample datasets, we adopted three approaches to simulate the multiomics cancer dataset:

1. Homogeneous multiomics cancer dataset: We randomly extracted a specific sample size from the 100,000 samples to obtain the data as X′, SNP'. Subsequently, Y′ was generated based on X′, SNP', and a predefined *θ.*
2. Low-heterogeneity multiomics cancer dataset: We maintained the same parameter settings as the simulation of the GWAS dataset but regenerated the multiomics cancer dataset with a certain sample size.
3. High-heterogeneity multiomics cancer dataset: We altered the association (*γ*) between SNP and **X** from a uniform distribution ranging from 0.08 to 0.10 to a truncated normal distribution (truncation at 0.08 and 0.10). The dataset was then regenerated with a certain sample size. This change increased variability in the strength of associations, thereby enhancing the population heterogeneity between the small-sample multiomics cancer dataset and GWAS.

Considering the sample sizes commonly used in multiomics cancer research, we established simulation sample sizes of 50, 100, 200, and 300, respectively. The true causal coefficient (*θ*) between the exposure **X** and the outcome **Y** was established under two scenarios: (1) when there was a certain causal relationship between **X** and **Y**, *θ* was assigned to 0.12 and 0.60 and (2) when there was no causal relationship between **X** and **Y**, *θ* was set to 0.

After generating individual-level data, we performed univariate linear regression analyses where each SNP served as the independent variable and the exposure/outcome as the dependent variable. This approach allowed us to derive summarized data, including regression coefficients and corresponding standard errors for SNP–exposure and SNP-outcome. SNPs that exhibited a significant association with the exposure, as supported by both the multiomics cancer simulation dataset (*p* < 0.05) and external GWAS data (Bonferroni-adjusted *p* < 0.05, consistent with GWAS findings), were selected as IVs. Leveraging the summary statistics derived from the multiomics cancer simulation dataset, we then implemented the inverse variance weighted (IVW) method to assess the causal effect of **X** on **Y**. The simulation study was conducted under a one-sample MR setting, and each setting was replicated 2000 times. All simulations were performed in R, and the code can be found at https://github.com/Li-Lab-SJTU/Phoslink/tree/main/Simulations.

### Proteomic and Phosphoproteomic Data From CNHPP-LUAD

A total of 79 cases of LUAD tumors and their paired noncancerous adjacent tissues (NATs) from treatment-naive Chinese patients were included in this study. The collection of proteomic and phosphoproteomic data followed the MS-based label-free quantification strategy of CNHPP (20). LC–MS/MS-generated MS raw files were searched against the UniProt human proteome database *via* MaxQuant software (version 1.6.5.0; The Max Planck Institute of Biochemistry) equipped with the Andromeda search engine. Intensity-based absolute quantification was utilized for quantifying both proteins and phosphosites. Intensity-based absolute quantification values calculated by MaxQuant were quantile normalized and log2 transformed if necessary (21). Detailed data preprocessing steps were outlined in a previous publication by our research group (8).

### Identification and Annotation of Germline SNPs

The detection of germline SNPs from the WES clean data of 79 NATs followed the GATK Best Practice Guidelines (version 4.2.0.0) (https://gatk.broadinstitute.org/hc/en-us/articles/360035535932-Germline-short-variant-discovery-SNPs-Indels). Initially, the clean reads were aligned to the human genome reference (GRCh38 assembly) using BWA MEM (version 0.7.17-r1188) with default parameters. The resulting alignment files were then sorted and indexed using Samtools (version 1.12), and PCR duplicates were marked using GATK MarkDuplicates. To ensure data quality, base quality recalibration was performed using the GATK BaseRecalibrator and ApplyBQSR modules. Variants were called from the recalibrated bam files using the GATK HaplotypeCaller module, and the resulting genomic variant call format (GVCF) files from the 79 sequenced individuals were combined using CombineGVCFs. Genotyping of the combined file was accomplished using GenotypeGVCFs. High-quality variant calls were retained through the VariantRecalibrator and ApplyRecalibration steps. During the VariantRecalibrator process in SNP mode, the model was trained using four standard SNP sets: HapMap3.3 SNPs, dbSNP build 146 SNPs, 1000 Genomes Project SNPs from Omni 2.5 chip, and 1000 G phase1 high-confidence SNPs. Variants were filtered using a sensitivity setting of 99.0% (--ts_filter_level 0.99) for SNPs. Subsequently, additional filtering was applied to the variants using vcftools (version 0.1.13), including criteria biallelic SNPs, autosomal SNPs, genotype calling rate >90%, and minor allele frequency >0.01 (22). Variants located in sex chromosomes were routinely excluded because of elevated noise in X-linked data (23) and low marker coverage in Y-specific regions (24). To enhance the accuracy of primary genotypes obtained using GATK, haplotype phasing and imputation were performed using Beagle (version 5.3; Brian Browning) (25) to impute sporadically missing sites. Finally, variants were annotated with gene context information using SnpEff (version 4.3; Pablo Cingolani) (26) transformed genotypes into 0 (homozygous wildtype), 1 (heterozygous), and 2 (homozygous mutant).

### Phoslink Screening for Causal Associations Between Phosphorylation and Protein Abundance

Phosphorylation can be influenced by DNA variants, either local or distant to their corresponding genes, known as *cis*- and *trans*-SNPs, respectively. *Cis*-SNPs are often considered ideal IVs for MR because of their larger effect sizes and highly plausible biological relationships with phosphorylation levels, as *cis*-acting DNA variants influence signaling regulation *via* protein phosphorylation, which in turn affects downstream changes like protein translation (27, 28). The IVs employed in the analyses were *cis*-SNPs (up to 1 Mb upstream or downstream), which minimized the risk of bias from horizontal pleiotropy. Then we filtered IVs further with the prior phosphorylation-related SNPs from the PhosSNP 1.0 database (16). Applying our CNHPP-LUAD proteomics dataset, we examined the associations of these *cis*-acting phosphorylation-related SNPs and phosphosites using a linear regression model adjusted for age, sex, and smoking status. SNPs that demonstrated significant association with the phosphosite (*p* < 0.05) served as IVs finally. When there was linkage disequilibrium between SNPs, a reference panel from the 1000 Genome Project (29) was used to remove linked SNPs at R^2^ >0.2, retaining the most significant SNPs (with the smallest *p* value) and ensuring independence between SNPs (30, 31, 32). Both the proteomic and phosphoproteomic datasets were logarithmically transformed before linear regression analysis (21). The beta coefficient (β) and standard deviation (ϵ) in the linear regression later were used as inputs to MR. Using the summary statistics derived from the multiomics cancer dataset, we applied the IVW method in the MendelianRandomization R package (33) to evaluate the causal effect of phosphorylation on protein expression.

$$XY=\beta_{0}+\beta \times G+\beta_{1}\times age+\beta_{2}\times sex+\beta_{3}\times smoking\;status+\epsilon$$

where **XY** can denote either exposure **X** (phosphorylation) or outcome **Y** (protein expression), *β*_*0*_ represents the intercept, *β* is the regression coefficient for the genotype **G**, *β*_*1*_, *β*_*2*_, and *β*_*3*_ are the corresponding regression coefficients for the covariates, and ε is error terms.

To ensure statistical power during MR analysis, we initially removed phosphosites and proteins with a missing value frequency of more than 90% and excluded SNPs with variation rates (proportion of nonhomozygous wildtype genotypes) below 10%. To reduce false-positive risk, only those phosphosites and proteins that showed statistically significant associations were retained (*p* < 0.05, Pearson’s correlation test).

### Association Between Biomolecule and Clinical Features

The Wilcoxon signed-rank test was utilized to examine whether biomolecules (phosphosites or proteins) were differentially expressed between the tumors and matched NATs. To identify biomolecules exhibiting differential expression across different stages, the Kruskal–Wallis test was performed. To explore biomolecules associated with overall survival (OS) or disease-free survival, Kaplan–Meier survival analysis was conducted, along with the log-rank test and Cox proportional hazards regression. Before the log-rank test, the optimal cutpoint for sample selection was determined using the survminer R package (version 0.4.9) with the maxstat (maximally selected rank statistics). To account for multiple comparisons, *p* values were adjusted using the Benjamini–Hochberg (BH) method. Both the proteomic and phosphoproteomic datasets underwent logarithmic transformation before analysis.

### Phosphoregulatory Network Analysis

Phoslink conducted a screening for causal associations between phosphosites (exposure) and proteins (outcome) using the PhosSNP and CNHPP-LUAD dataset. Subsequently, a phosphoregulatory network was constructed based on these causal phosphosite–protein links. The functionality of phosphoregulators was assessed by quantifying the similarity between their downstream proteins and established cancer hallmarks (34) through GOSemSim (version 2.26.0) (35). Pairwise semantic similarities were computed using the graph-based similarity measure algorithm proposed by Wang *et al*. (36), which determines the semantic similarity of two GO (Gene Ontology) terms based on their locations in the GO graph and their relations with ancestor terms. This method encodes the semantics (biological meanings) of GO terms into a numeric value by aggregating the contributions of their ancestor terms. Semantic comparisons of GO annotations provide a quantitative measure of functional similarities between gene groups based on these data. Each phosphoregulator was annotated to the hallmark exhibiting the highest semantic similarity score with the clusterSim.

### Druggability Assessment

We first collected information on drug targets from Drug–Gene Interaction Database 4.0 (37), DrugBank (38), and Therapeutic Target Database (39) to explore their potential as known drug targets for drug repurposing in LUAD treatment. To assess the druggability of phosphoregulators, we investigated their presence within functional domains annotated in the InterPro database (40), utilizing InterProScan with default settings to search protein sequences for potential matches against InterPro protein signature databases. In addition, given the pivotal role of kinases as promising therapeutic targets in cancer (3), we gathered experimentally validated human kinase–substrate relationships from the Phospho.ELM (41), PhosphoSitePlus (42), and PhosphoNetworks (43) databases.

### Estimation of Causal Mediation Effects

We conducted mediation analyses to assess the proportional contribution of regulatory phosphosites to LUAD clinical features using the R mediation package (version 4.5.0) (44). The analysis consisted of two steps. In the first step, we developed two statistical models, a mediator model for the conditional distribution of the mediator (protein) given the treatment (phosphosite) and an outcome model for the conditional distribution of the LUAD clinical phenotype (survival data). We fitted these models separately and used the resulting fitted objects as inputs for the mediate function, which calculated the effect mediated by the protein, effect unmediated by the protein, and total effect. We fitted the mediator and outcome models to the observed data using the lm and survreg functions from the R stats and survival packages, respectively. These models included age, sex, and smoking status as covariates. The mediation analysis model was executed using 1000 simulations with a quasi-Bayesian approach to estimate confidence intervals.

## Results

### An Overview of Phoslink

**Fig. 1:**
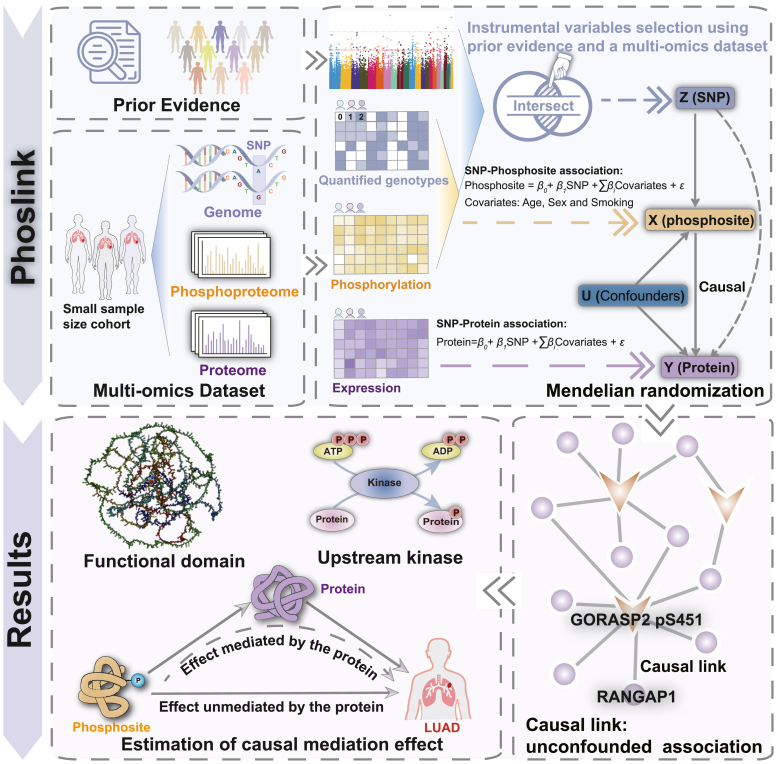
**The analytic framework of the study.** To characterize the functionality of identified phosphosites in multiomics cancer research, we introduce a causal inference model termed “Phoslink,” which integrates prior evidence and a multiomics cancer dataset to infer causal regulatory links between protein phosphorylation and expression. Applied to the CNHPP-LUAD dataset, Phoslink uncovered regulatory links and identified key phosphosites, including MAP4 pS941, located within a therapeutic target domain in lung carcinoma. A detailed mediation analysis further assesses the impact of phospho-based regulation on survival, distinguishing between effects mediated by proteins and those that are not. CNHPP, Chinese Human Proteome Project; LUAD, lung adenocarcinoma.

We present a novel approach to identify the *Phos*phoregulatory *link* through causal inference, named Phoslink. Because of the dynamic nature of protein expression and phosphorylation status, as well as the notable heterogeneity across different populations and cellular states, one-sample MR is more appropriate to infer causal relationships between protein expression and phosphorylation. Extensive proteogenomic data in CPTAC and CNHPP provide opportunities for Phoslink to infer the causal effects of phosphorylation on protein expression with a one-sample MR setting. This approach could provide a more reliable and robust causal inference, considering the changes in protein expression and phosphorylation and the genetic variation among individuals within the same population. Given that the existing IV selection methods are primarily used for studies with large sample sizes, the criteria for IV selection in the context of small-sample multiomics cancer data are more stringent. It is important to note that when dealing with sample sizes small, substantial effects may result in seemingly insignificant *p* values (45, 46). Such stringent thresholds may result in only a small subset of X being eligible as IVs for causal estimation analysis. Phoslink adopts a more relaxed threshold setting based on a small-sample phosphoproteomics dataset and incorporates external prior evidence as auxiliary screening: (1) Internal data support, indicating that the SNP–exposure relationship is statistically significant based on the small-sample dataset (*p* < 0.05 from a linear regression model adjusted for age, sex, and smoking status) and (2) External prior evidence, where prior studies provide support for the SNP–exposure relationship (Fig. 1). The PhosSNP 1.0 database (16) serves as a source of phosphorylation-related SNPs for external prior evidence in IV selection (47). SNPs that meet both criteria are selected as IVs, thus considering the heterogeneity of cancer data and effectively reducing false-positive rates. Next, we estimated the associations of each IV with the exposure and outcome using beta-coefficients and their standard errors from linear regression analyses. For the primary MR analysis, we applied IVW regression method in the MendelianRandomization R package (version 0.6.0) (33). We used the IVW two-sample MR method because the IVW method offers robust causal estimates by combining ratio estimates of each variant within a fixed-effects meta-analysis model (48) and is regarded as a safe option for one-sample MR analyses (49, 50). Phoslink is freely available at https://github.com/Li-Lab-SJTU/Phoslink.

### Feasibility and Performance of Phoslink

**Fig. 2:**
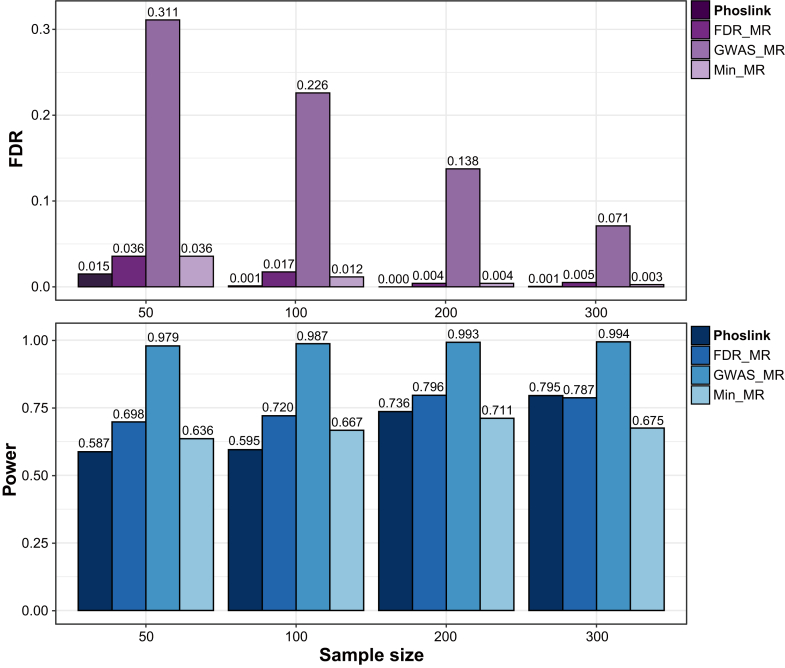
**Performance evaluation of Phoslink.** FDR (true causal effect *θ* = 0) and Power (true causal effect *θ* = 0.6) for Phoslink and other methods in simulations at different sample sizes. The *x*-axis shows the sample size, whereas the *y*-axis displays the FDR (*purple*) or power (*blue*) for each method. FDR, false discovery rate.

To assess the reliability of Phoslink, we compared Phoslink against three IV selection strategies in MR analyses, which are frequently utilized in studies with large sample sizes: (1) FDR_MR: which selects all SNPs significantly associated with X (BH-adjusted *p* < 0.05) based on the simulated small-sample multiomics cancer dataset; (2) Min_MR: which selects the SNP with the minimum *p* value among the SNPs significantly associated with X (BH-adjusted *p* < 0.05) based on the simulated small-sample multiomics cancer dataset; and (3) GWAS_MR: which selects SNPs significantly associated with X as supported by the simulated external GWAS studies study (Bonferroni-adjusted *p* < 0.05). We conducted a comprehensive comparison of the power (with a true causal effect, *θ* = 0.60) and FDR (assuming a null causal effect, *θ* = 0) across four distinct methods. Based on the simulated evaluation results of a homogeneous multiomics cancer dataset, Phoslink displayed a lower FDR while maintaining competitive power compared with other methods (Fig. 2). These results remained robust in sensitivity analyses employing diverse proportions of invalid IVs including 30% and 50% (Supplemental Fig. S2, *A* and *B*). In addition, in two simulated datasets with varying degrees of heterogeneity, Phoslink consistently demonstrated similar performance, exhibiting comparable power while effectively reducing the FDR (Supplemental Fig. S2, *C* and *D*). To assess the robustness of the Phoslink method, we performed simulations by randomizing SNP identities across the small-sample dataset while maintaining its structure, repeating this process 2000 times for reliability. The results showed a significant decrease in Phoslink’s accuracy with varying sample sizes, with power metrics around 0.5 regardless of sample size (Supplemental Fig. S3).

**Fig. 3:**
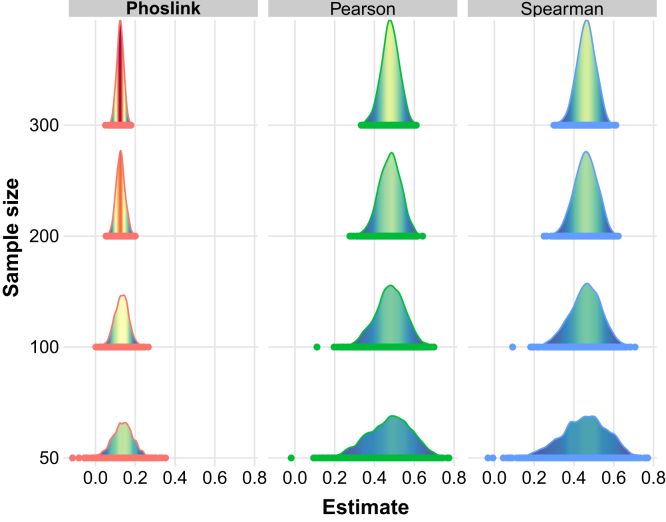
**Comparison of accuracy and consistency between Phoslink and correlation analyses.** Density plots of effect estimates from Phoslink, Pearson, and Spearman analyses with 0% invalid IV scenario. The *x*-axis represents the estimates from the three methods. IV, instrumental variable.

Next, we compared the results from Phoslink and conventional correlation analysis, namely Pearson and Spearman correlation commonly used to elucidate molecular associations in cancer research. Under the assumption of a causal effect of 0.12 and the genetic effects of SNPs on the exposure drawn from a uniform distribution *U*(1.0, 1.5), Phoslink exhibited lower variability in causal estimation and yielded a more robust and precise estimate of effect size compared with Pearson and Spearman (Fig. 3 and Supplemental Fig. S4). Moreover, when the effect size was set to 0, Phoslink presented a reduction of FDR, and this advantage became more pronounced as the sample size increased (Table 1). These findings collectively support the validity and reliability of Phoslink as a valuable tool for detecting causal relationships among multidimensional biomolecules.

**Table 1:** FDR (true causal effect *θ* = 0) for the three methods at different sample sizes

Sample size	Phoslink	Pearson	Spearman
0% invalid IV			
50	0.0095	0.0015	0.0005
100	0.0030	0.0030	0.0040
200	0	0.0370	0.0260
300	0	0.1450	0.1130
30% invalid IV			
50	0.0125	0.0005	0.0005
100	0.0085	0.0065	0.0080
200	0.0015	0.1130	0.0885
300	0	0.2675	0.2110
50% invalid IV			
50	0.0105	0.0005	0.0005
100	0.0195	0.0110	0.0100
200	0.0140	0.1155	0.0880
300	0.0045	0.2550	0.2240

### Characterization of Germline SNPs in LUAD

**Fig. 4:**
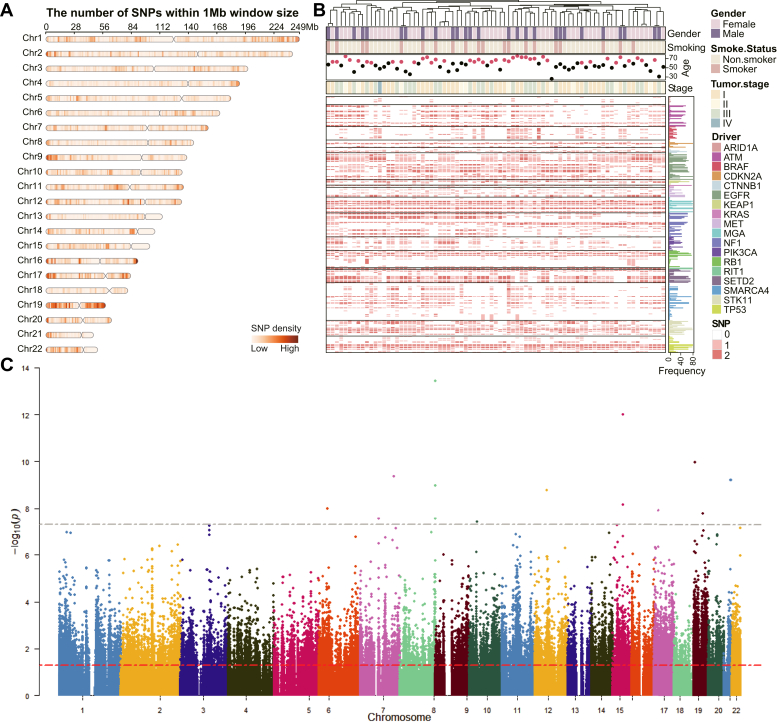
**Overview of detected germline SNPs in LUAD.***A*, distribution of germline SNPs on autosomal chromosomes within a 1 Mb window size. The *light color* represents a low content, and the *dark color* represents a high content of germline SNPs. *B*, profiling of germline SNPs in the well-defined driver genes of LUAD. *Rows* correspond to germline SNPs in LUAD driver genes, and *columns* represent the 79 samples, showing the mutation status: 0 (homozygous wildtype), 1 (heterozygous genotype), and 2 (homozygous mutant). *C*, Manhattan plot for all phosphosites reveals *p* values from univariable linear regression adjusted for age, sex, and smoking status and germline SNP positions across 22 autosomal chromosomes. The *horizontal lines* indicate the genome-wide cutoff of 5 × 10^−8^ (*gray*) and 0.05 (*red*), respectively. The *y*-axis shows the -log10 of the *p* values for the associations of genetic variants with phosphorylation levels. LUAD, lung adenocarcinoma.

WES data from CNHPP-LUAD cohorts resulted in a total of 310,576 autosomal germline SNPs with a major allele transition from C to T (Supplemental Fig. S5*A*), and chromosome 1 exhibited the highest number of SNPs (Supplemental Fig. S5*B*). The germline SNPs were not evenly distributed among chromosomes or within chromosomes (Fig. 4*A*), depending on the guanine–cytosine content of the chromosomes (51). The observed Ti/Tv ratios in our dataset were in agreement with the expected ratio, with an overall Ti/Tv ratio for all samples being 2.34. Among the 20 well-defined driver genes of LUAD (52), *SMARCA4* and *EGFR* displayed the highest SNP counts, with *SMARCA4* possessing 50 SNPs and *EGFR* containing 36 SNPs (Fig. 4*B* and Supplemental Fig. S5*C*). In contrast, *RBM10* and *U2AF1* showed no SNPs within their sequences.

The collection of SNPs, proteomics, and phosphoproteomic data in CNHPP-LUAD cases enables Phoslink to infer the causal effects of phosphorylation on protein expression. Initially, we investigated the association of each phosphosite (exposure) and *cis*-acting phosphorylation-related SNPs (IV candidates) in 79 LUAD tumors using a linear regression model adjusting for age, sex, and smoking status. In total, there were 1,240,601 pairs of SNP–phosphosite associations involving 7910 phosphosites and their corresponding 220,923 *cis*-SNPs. However, only 11 associations reached genome-wide significance (*p* < 5 × 10^−8^) (Fig. 4*C*). A substantial number of SNPs did not reach this stringent significance level but still deserved further investigation as large effects may produce unimpressive *p* values if the sample size is small (45, 46). For instance, the rs13407823 genotype was significantly associated with IWS1 phosphorylation level (Kruskal–Wallis test *p* < 0.001) (Supplemental Fig. S5*D*). Individuals who were homozygous for the minor allele (G/G) at rs13407823 were found to be associated with a decreased level of phosphorylation compared with homozygosity for the reference allele (A/A). Similarly, the phosphorylation level of SRRM1 was significantly lower in patients carrying the T allele for rs55691364 (Kruskal–Wallis test, *p* < 0.001) (Supplemental Fig. S5*E*). The threshold for IV selection in the context of small-sample multiomics cancer data is more stringent, dramatically lessening the range of potential causal relationships that can be evaluated. FDR_MR and Min_MR, which use more lenient thresholds, showed that only 51% (879/1727) and 66% (638/966) of their identified associations, respectively, were significantly correlated, suggesting that a substantial proportion of these associations may not represent true biological relationships. In addition, correlation-based methods like Pearson and Spearman identified a large number of phosphosite–protein links, but these were prone to high false-positive rates because of confounders.

### Causal Inference of Phosphoregulatory Network

**Fig. 5:**
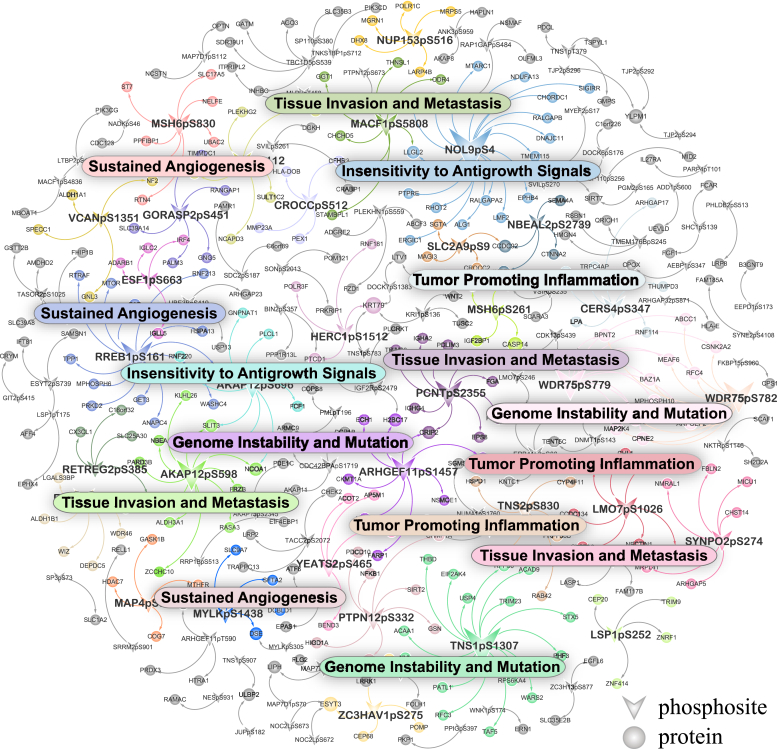
**Phosphoregulatory network in LUAD.** The network indicates the regulatory relationship between phosphoregulators and proteins. Node colors correspond to the most closely related cancer hallmarks, determined by the similarity of the protein sets regulated by phosphoregulators (limited to those regulating six or more proteins), and node size is proportional to node degree. LUAD, lung adenocarcinoma.

To identify phosphorylation-related SNPs among millions of SNPs in GWAS datasets, we downloaded a list of such SNPs from the PhosSNP 1.0 database (16), containing 64,035 entries specific to human phosphorylation events. Phoslink screened for causal associations between phosphosite (exposure) and protein (outcome) based on the PhosSNP and CNHPP-LUAD dataset. A total of 345 significant phosphosite–protein links were identified in the CNHPP-LUAD dataset. We introduced noise by randomizing SNP identities within the dataset. The overlap between the relationships identified in the perturbed data and those in the original dataset was only one, demonstrating the advantage of our method in specificity. The 345 casual links encompass 109 regulatory phosphosites (phosphoregulators) and 310 proteins (BH-adjusted *p* < 0.05). We observed that there was a strong correlation between the magnitudes of the causal estimates and the observational correlation coefficients (*r* = 0.71 with Pearson, *r* = 0.66 with Spearman), and the signs of the MR estimates were generally in the same direction as the Pearson correlation coefficients, with a 100% match for Pearson and 98.84% match for Spearman (1.16% discrepancy, 4/345). Subsequently, we constructed a phosphoregulatory network based on 345 causal links (Fig. 5). We assessed the functionality of phosphoregulators by quantifying the similarity between their downstream proteins and established cancer hallmarks (34) utilizing the GOSemSim (35). Functional roles of these phosphoregulators were inferred based on the hallmark exhibiting the highest semantic similarity score.

**Fig. 6:**
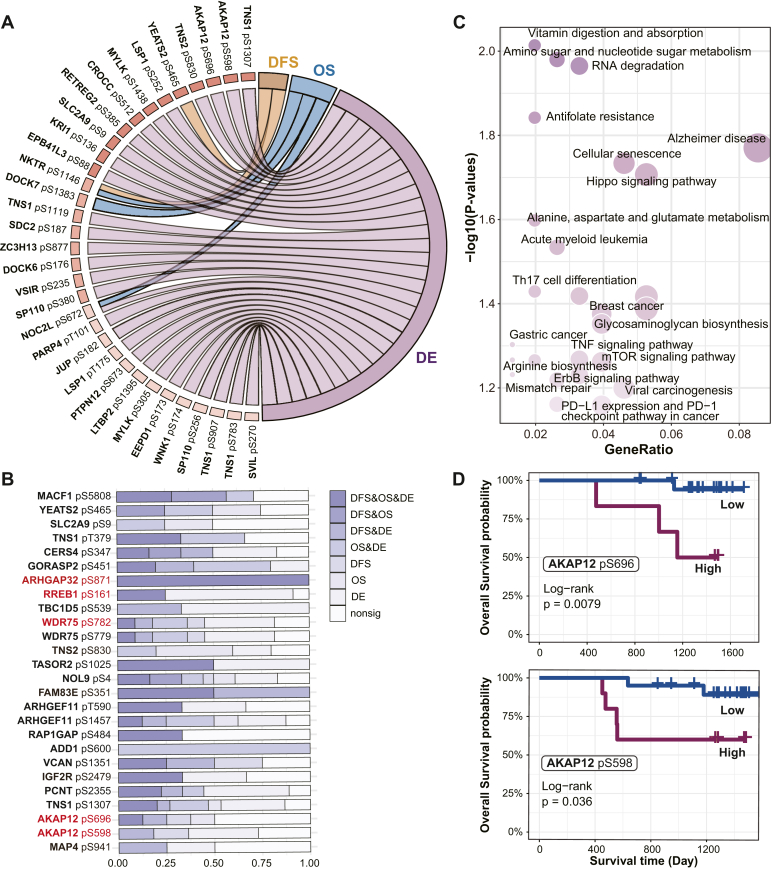
**Exploring the significance of regulators in LUAD.***A*, distribution of the regulators associated with different clinical features in LUAD (FDR <0.05). *B*, stacked histogram showing the distribution of proteins associated with different clinical features regulated by 26 key regulators. The *red-labeled* key regulators are significantly associated with clinical features in two independent LUAD datasets.*C*, the dot plot describes the KEGG pathway enrichment for all key regulators, with dot size scaled by the GeneRatio, and color denoting the significance of association. *D*, Kaplan–Meier curves of overall survival, categorized by low *versus* high AKAP12 at S696 and S598 phosphorylation levels. FDR, false discovery rate; KEGG, Kyoto Encyclopedia of Genes and Genomes; LUAD, lung adenocarcinoma.

To explore the impact caused by phosphoregulators on the clinical features, we initially connected their phosphorylation signals with four LUAD clinical features: tumor stage, OS, disease-free survival, and differential phosphopeptide abundances between tumor and normal samples (differential analysis). Among the 109 phosphoregulators (see Supplemental Table S1 for the full list), 30.28% (33/109) phosphoregulators demonstrated significant association with LUAD clinical features (Fig. 6*A*). In further analysis, we checked the associations between the downstream proteins regulated by phosphoregulators and the features. We identified the 26 phosphosites, involving 22 proteins, as key phosphoregulators, each linked to at least one downstream protein significantly associated with LUAD clinical features, including survival and differential analysis (Fig. 6*B*). Pathway enrichment analysis identified several well-recognized oncogenic pathways among the top pathways enriched by these downstream proteins (Fig. 6*C*), such as the Cellular senescence, mTOR signaling pathway, and various metabolic pathways. Moreover, several immune-related pathways were observed, such as PD-L1 expression and PD-1 checkpoint pathway in cancer. Notably, 16 of these key phosphoregulators were exclusively identified through our Phoslink model. Among them, AKAP12 undergoes hyperphosphorylation on serine residues during the G1/S phase. This serine phosphorylation, regulated by PKC and other kinases, alters the interaction of AKAP12 with the cytoskeleton matrix, thereby controlling critical processes, such as cytokinesis, cell proliferation, and cell migration (53, 54). In the CNHPP-LUAD cohort, elevated phosphorylation levels of AKAP12 at S696 and S598 showed significant associations with poor survival (Fig. 6*D*). Several additional key phosphoregulators have also been reported to play significant functional roles, including RREB1 pS161 (55, 56) and MAP4 pS941 (57). Besides, while certain proteins currently lack functional validation at present sites, their phosphorylation levels indisputably hold crucial functional significance, such as ADD1 (58, 59), PCNT (60), RAP1GAP (61) and TNS1 (62). This emphasized the capability of Phoslink to uncover novel key regulatory phosphosites, adding to the current repertoire of biomolecular understanding of LUAD.

Next, we assessed the clinical relevance of the 26 phosphoregulators in two independent LUAD proteome cohorts: one from the United States (CPTAC-LUAD) (47) and the other from East Asian populations (Taiwan-LUAD) (63). Our results showed that, of the 26 key phosphoregulators identified in the CNHPP-LUAD cohort, nine were detected in the CPTAC-LUAD cohort. Relatively fewer shared phosphosites across cohorts were not detected because of technical and population-related factors (64). Eight of these nine were consistently associated with clinical phenotypes, of which three exhibited statistical significance in both differential analyses between cancer and adjacent tissues, and survival analyses (FAM83E pS351, RREB1 pS161, and TNS2 pS830). In the Taiwan-LUAD cohort, 16 of the 26 phosphoregulators were detected (missing value rate <0.9), with 13 showing statistically significant differences between cancer and adjacent tissues (one-sample Wilcoxon signed-rank test based on Supplemental Table S1*G*_PhosSiteLog2TN in the study, *p* < 0.05). Among these 13, TASOR2 pS1025 and WDR75 pS782 also exhibited significant differences between tumor stages (stage 1 *versus* others).

### Functional Roles of Key Phosphoregulators in LUAD

Integrating phosphoregulators with data from drug databases could help identify potential drug candidates for repurposing in LUAD treatment. Previous studies have demonstrated that compounds targeting proteins supported by genetic evidence are more likely to work than those without such support (65). Among the 22 proteins harboring 26 key phosphoregulators, four have already been identified as drug targets, including IGF2R, VCAN, MAP4, and ADD1 according to the Drug–Gene Interaction Database (37), DrugBank (38) and Therapeutic Target Database (39).

**Fig. 7:**
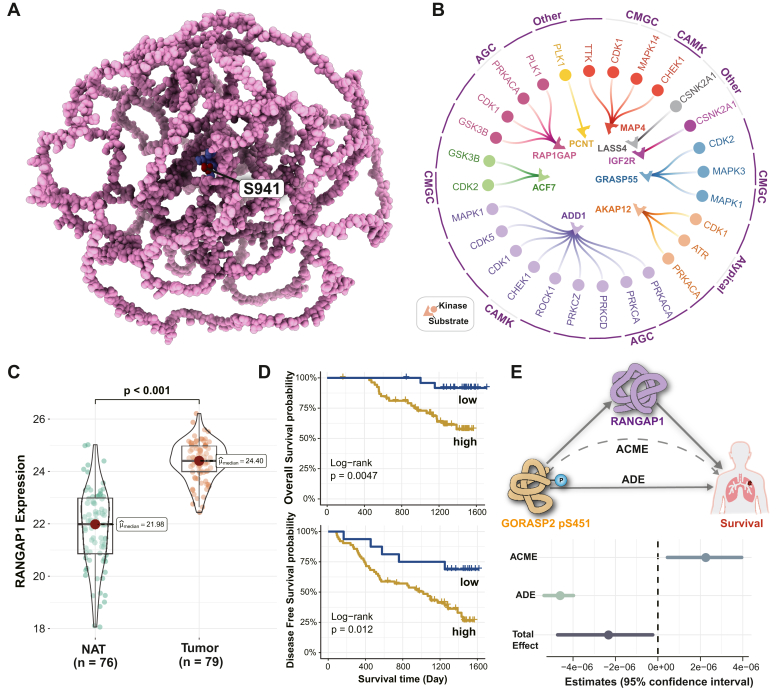
**Evaluating the potential of regulators for drug development.***A*, the 3D structure of MAP4 with pS941 marked with *red* using UCSF’s Chimera X visualization tool, where *blue* indicates the tubulin-binding domain. *B*, network diagrams depict the upstream kinases of phosphoregulators, and the outermost layer presents the group information of kinases. *C*, protein expression of RANGAP1 in tumors *versus* NATs, with the Wilcoxon signed-rank test *p* value indicated on *top*. *D*, Kaplan–Meier plots illustrating overall and disease-free survival in samples from the LUAD cohort, categorized by low *versus* high RANGAP1 protein expression. High expression of RANGAP1 is significantly associated with worse outcomes. *E*, mediation analysis quantified the effect sizes of the RANGAP1 mediator model, with GORASP2 pS451 as the exposure and LUAD survival as the outcome. LUAD, lung adenocarcinoma; NAT, noncancerous adjacent tissue.

To better understand the structural properties of the prioritized phosphoregulators, we first investigated whether these phosphosites reside within functional domains annotated in the InterPro resource (40). Notably, 88.46% (23/26) of the phosphoregulators were located within known InterPro domains. For instance, the MAP4 pS941 is situated within the tubulin-binding domain, as visualized in UCSF ChimeraX (66) (Fig. 7*A*). It is noteworthy that Paclitaxel, a tubulin-binding agent, is a commonly used first-line therapy for non–small-cell lung carcinoma (67). We calculated the site-specific disorder score using the IUPred3 (68) and classified scores exceeding 0.5 as disorder. The regulators are predominantly located in intrinsically disordered regions (Supplemental Fig. S6*A*). To further explore the possible functionalities of the phosphoregulators, we assigned a functional score to each, ranging from 0 to 1, based on the methodology by David Ochoa *et al*. (69). A higher score indicates a higher likelihood of relevance for cell fitness. The phosphosites identified by our algorithm have significantly higher functional scores (Supplemental Fig. S6*B*), regardless of whether the functional scores were directly obtained from Supplemental Table S3 of David Ochoa *et al*. (69) or recalculated using their tool, funscoR. Several phosphoregulators obtained a functional score higher than 0.4 (Supplemental Fig. S6*C*), such as RREB1 pS161, TBC1D5 pS539, and GORASP2 pS451, further emphasizing their functional significance. These findings support their candidacy as both biomarkers and drug targets. Considering the crucial role of kinases, as attractive therapeutic targets for cancer (3), we collected experimentally verified kinase–substrate relationships from Phospho.ELM (41), PhosphoSitePlus (42), and PhosphoNetworks (43). A total of 28 kinases were identified for the nine proteins harboring key phosphoregulators. Among them, ADD1 is targeted by the highest number of kinases with a total of nine (Fig. 7*B*). As expected, all these kinases are targeted by Food and Drug Administration–approved drugs, rendering them deserving further investigation into their potential therapeutic implications in LUAD.

To further elucidate how protein phosphorylation affects LUAD survival, we conducted mediation analyses. These analyses allowed us to decompose the total effect of phosphoregulators on survival into two components: the effect unmediated by the protein and the effect mediated by the protein. RANGAP1, a downstream protein of the key phosphoregulator GORASP2 pS451, was significantly associated with prognostic outcomes and showed elevated expression in tumors compared with NATs (Fig. 7, *C* and *D*). Mediation analysis revealed that GORASP2 pS451 exerted a significant mediation effect on survival by RANGAP1 protein expression (*p* = 0.018) (Fig. 7*E*). Interestingly, we observed opposing effects between the effect mediated by the protein and effect unmediated by the protein of GORASP2 pS451 on OS, which attenuated the total effect. This complexity in regulatory interactions may explain the difficulty in their identification using conventional methods.

## Discussion

The functional importance of protein phosphorylation has been established, yet the underlying mechanisms are still not well understood. Given the conventional stringent threshold for IV selection within multiomics cancer studies characterized by relatively small sample sizes, the majority of exposures remain unavailable for assessing potential causal relationships because of the lack of IVs. In this study, we introduced Phoslink, an MR-centered strategy designed for small-sample cancer datasets, achieving causal inference of phosphosite–protein links based on multiple omics data. Phoslink integrates both internal and external evidence to reduce the winner’s curse and allow the inclusion of weak instruments to the MR analysis for small-sample multiomics cancer data. We demonstrate the effectiveness of Phoslink across different simulation scenarios, showing its ability to reduce the FDR while maintaining comparable power to conventional IV selection methods like FDR_MR, Min_MR, and GWAS_MR, even in datasets with pronounced heterogeneity. Phoslink outperforms correlation-based methods, including Pearson and Spearman, by effectively reducing the FDR and providing more stable estimates for phosphoproteomic regulation within cancer research. Besides, the moderate correlation between the absolute magnitudes of the causal estimates and the observational correlation coefficients supports the previous report that even in estimating associations between “omic” variables, the use of causal inference methods is crucial for inferring causal effect sizes over observational associations (70). Phoslink is freely available on GitHub, ensuring accessibility to the wider scientific community and facilitating collaboration for further advancements in this field.

In our application of Phoslink to 79 LUAD samples, we identified over 300 significant phosphosite–protein pairs and constructed a comprehensive phosphoregulatory network. Analysis of the regulatory network revealed 26 phosphoregulators that hold promising potential as biomarkers associated with LUAD. These regulators exhibited significant enrichment within proteins related to LUAD clinical features and were involved in well-known oncogenic pathways. Moreover, several immune-related pathways were identified including PD-L1 expression and PD-1 checkpoint pathway in cancer, which may contribute to immune evasion of tumor cells (71). Importantly, 16 of 26 phosphoregulators were exclusively identified through phosphosite–protein causal relationships, reinforcing the value of causal inference in biomarker discovery. The novel regulators identified through our research not only provide fresh insights into the phosphosite–protein interactions in LUAD but also open new avenues for drug repurposing in LUAD treatment. MAP4 is a target of paclitaxel, docetaxel, and artenimol. Paclitaxel and docetaxel have established treatments in clinical practice for LUAD. Artenimol, an artemisinin derivative, is currently used against *Plasmodium falciparum* infection. However, numerous studies have indicated a close association between LUAD and the gut-lung microbiota (72) and the anticancer efficacy of artemisinin derivatives has been confirmed (73). Artenimol thus holds promising potential as a therapeutic option for LUAD. Importantly, MAP4 pS941, a key phosphoregulator we identified, resides within the tubulin-binding domain and paclitaxel is just a tubulin-binding agent. Several regulators showed their functional significance. Mediation analysis revealed that the opposing effects between the mediated and unmediated components by the protein of protein phosphorylation on OS, which ultimately attenuated the total effect, may account for the challenges in identifying these interactions using conventional methods.

While Phoslink demonstrates significant advancements in causal inference and annotation of phosphoproteomic data, it is important to acknowledge its limitations. First, MR analyses rely on three core IV assumptions for testing the causal effects of exposure on the outcome. These assumptions encompass the association of IVs with the exposure (relevance), their independence from potential confounders (independence), and their exclusive influence on the outcome only through the exposure and not through alternative pathways (exclusion restriction). DNA variants are not generally influenced by confounders based on the biological belief and they influence signaling regulation *via* protein phosphorylation, which in turn affects downstream changes like protein translation (27). Consequently, we assumed that the IV is associated with the exposure but not the outcome or any confounders in this study. However, in practice, fully ensuring the fulfillment of all these assumptions can be challenging, especially considering the possibility of pleiotropy in the biological context. This can potentially introduce bias in causal estimates. Second, Phoslink depends on the assumption of linear relationships among genetic variations, proteins, and phosphosites. Although this assumption simplifies analysis and interpretation, it may be not always accurate, given the nonlinear and dynamic nature of biological systems. Future research using nonlinear models may provide a more comprehensive and accurate understanding of molecular interactions. In summary, Phoslink emerges as a promising method for identifying causal phosphosite–protein links, thereby accelerating the clinical translation of cancer proteomics and phosphoproteomic data.

## Data Availability

The processed data are available at https://www.cell.com/cell/fulltext/S0092-8674(20)30676-0. Source data are provided in this article. We have implemented Phoslink in a computationally efficient R package that can be accessed at https://github.com/Li-Lab-SJTU/Phoslink. R code for reproducing the simulation data is available at https://github.com/Li-Lab-SJTU/Phoslink/tree/main/Simulations.

## Supplemental data

This article contains supplemental data.

## Conflict of interest

The authors declare no competing interests.
