# Supplementary material for: Causal Inference and Annotation of Phosphoproteomics Data in Multiomics Cancer Studies
Source: Mol Cell Proteomics. 2025 Jan 9;24(3):100905. doi: 10.1016/j.mcpro.2025.100905 (PMC11889353; doi:10.1016/j.mcpro.2025.100905)
Supplement: Supplementary information [file mmc1.pdf]

Supplementary Information

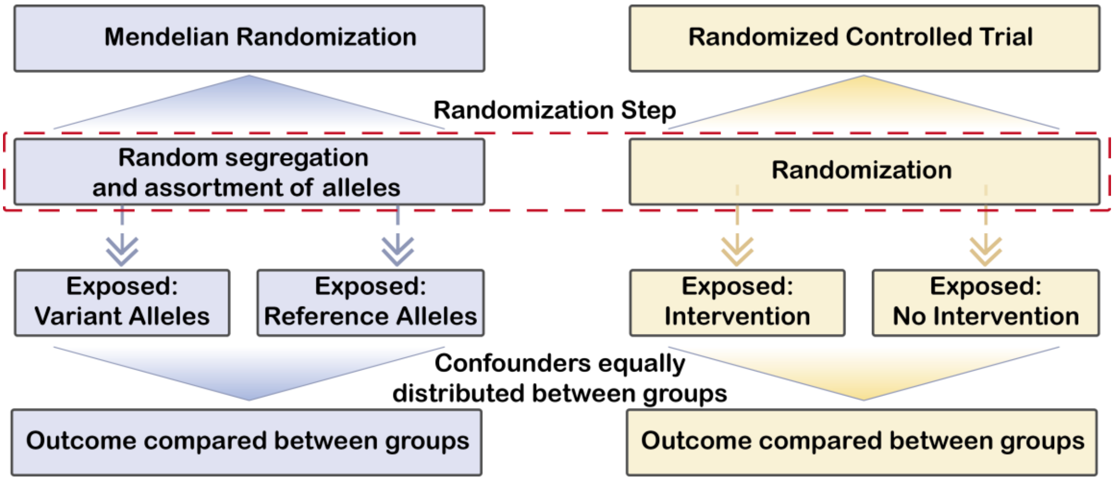

Figure S1. Comparison of Mendelian randomization (MR) with randomized controlled trial (RCT). In MR, randomization arises from the random allocation of alleles, similar to the randomization process in a controlled trial, where the assignment of alleles is independent of population-level variation.

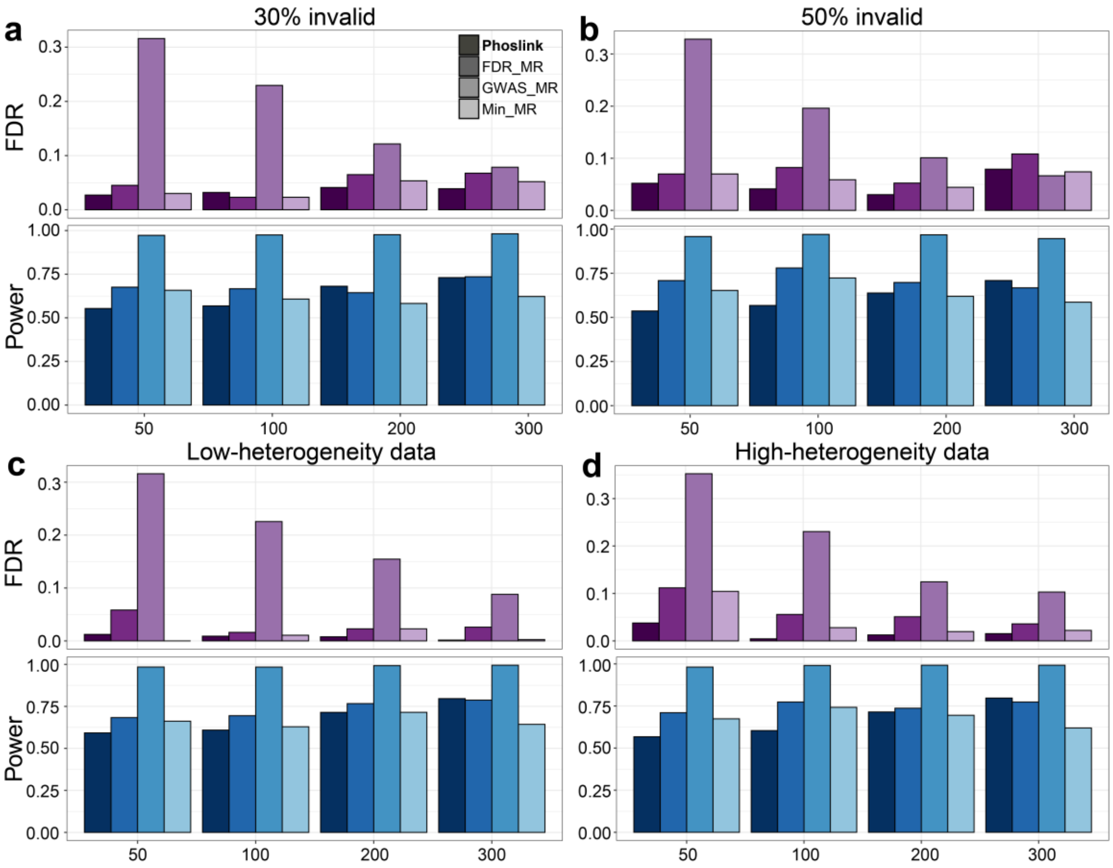

Figure S2. Power (θ = 0.60) and FDR (θ = 0) evaluation of Phoslink and other methods in simulations with 30% **(a)** and 50% **(b)** invalid instruments. Performance

assessment in low heterogeneous **(c)** and high heterogeneous **(d)** simulations at different sample sizes.

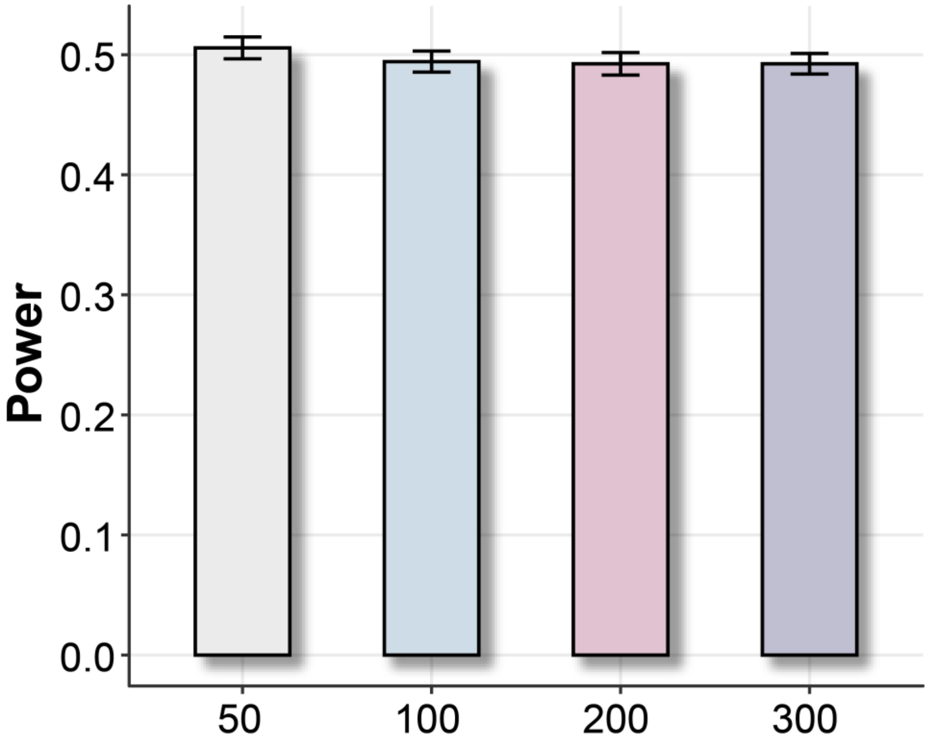

Figure S3. Power comparison of SNP randomization applied to the simulated small-sample dataset.

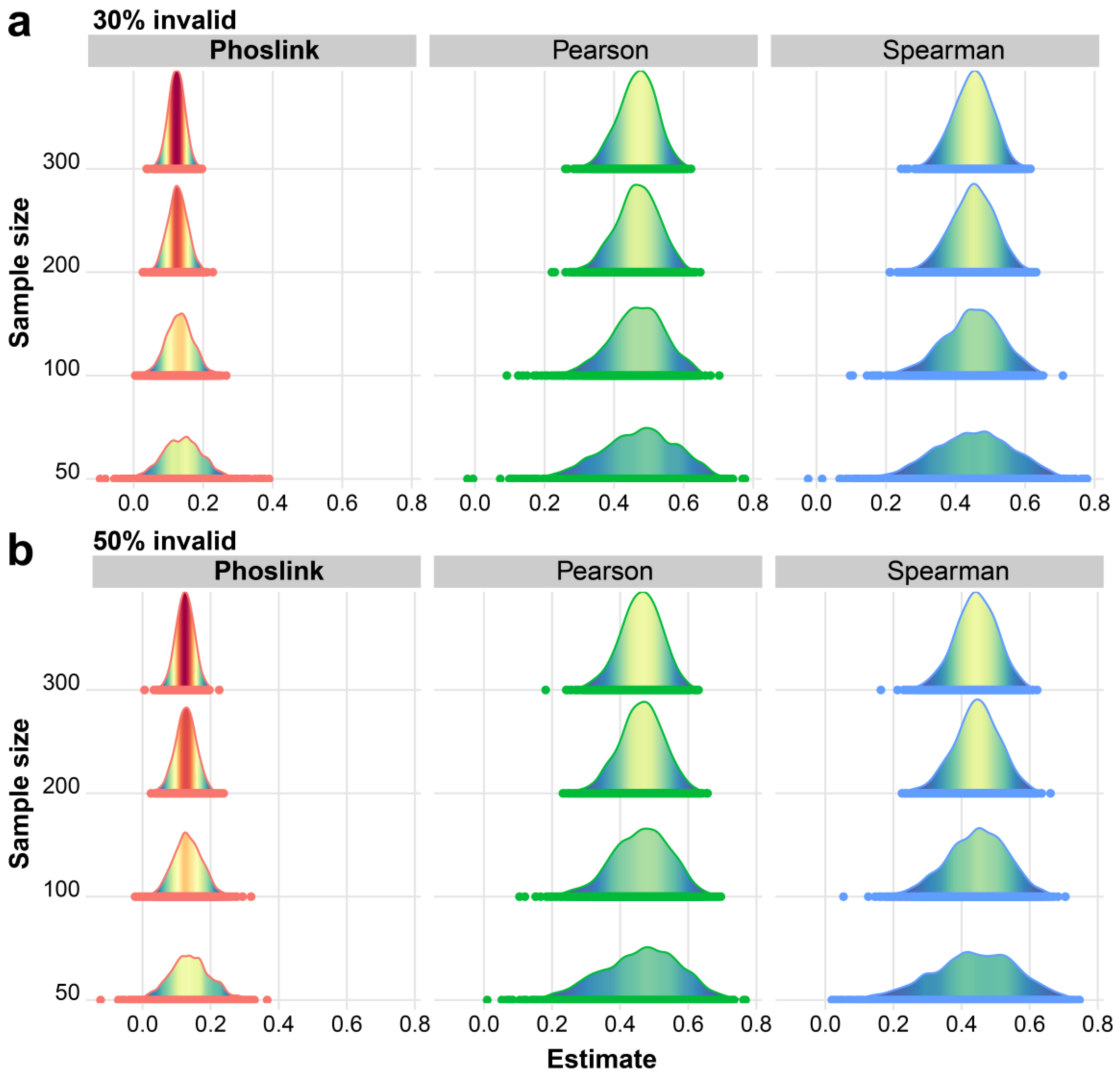

Figure S4. Density plots of effect estimates from Phoslink, Pearson and Spearman analyses with 30% **(a)** and 50% **(b)** invalid IVs scenarios.

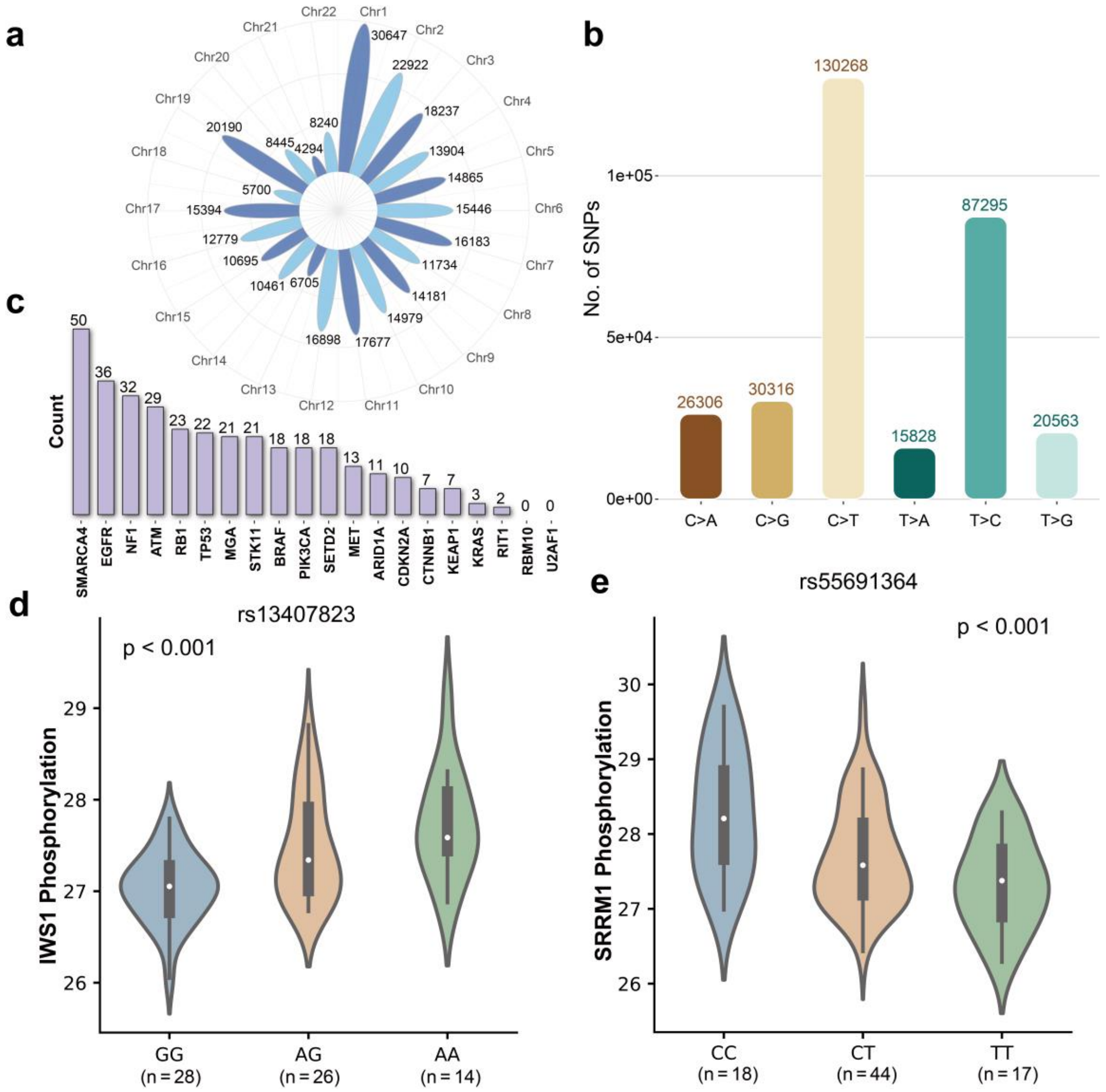

Figure S5. **(a)** Chromosomal distribution of germline SNPs. **(b)** Frequency of transition and transversion SNPs. **(c)** The number of germline SNPs across 20 well-defined driver genes of LUAD. Boxplots showing phosphorylation levels across germline genotypes for SNPs in **(d)** IWS1 (rs13407823; GG/AG/AA) and **(e)** SRRM1 (rs55691364; CC/CT/TT). P values were determined by Kruskal-Wallis tests.

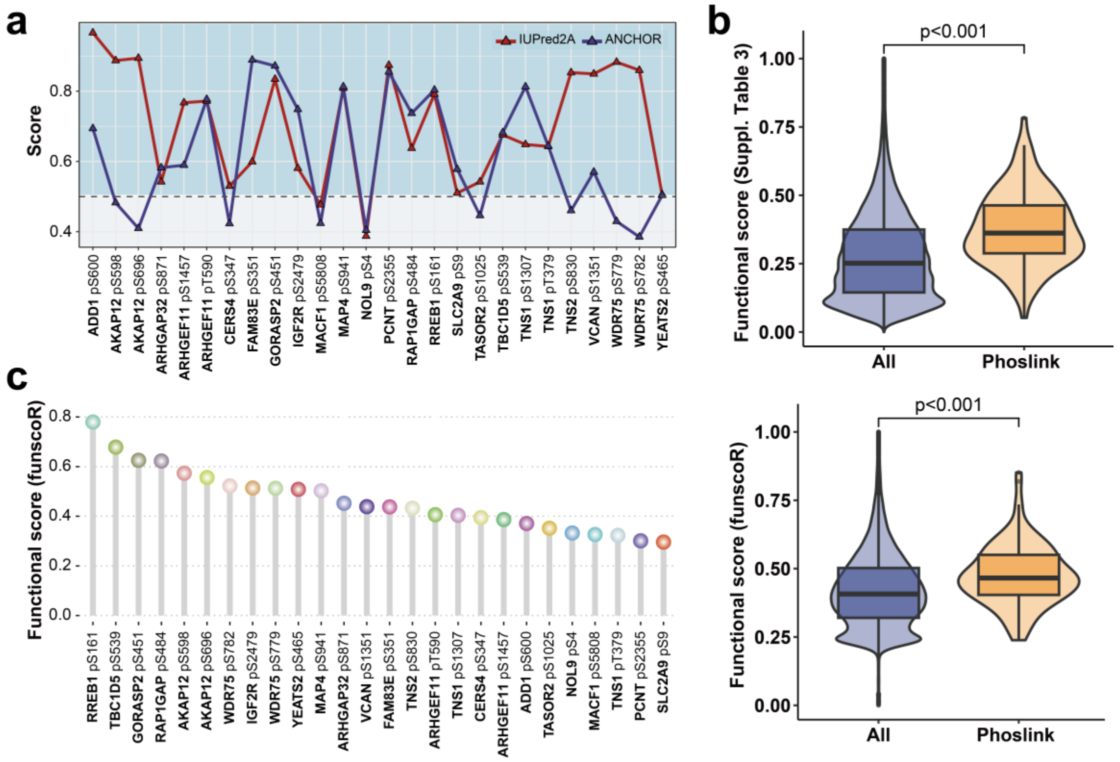

Figure S6. (a) Prediction of intrinsically disordered regions (IUPred2) and disordered binding regions (ANCHOR2) for phospho-regulators, highlighted in red and blue, respectively. Regions with scores > 0.5 indicate disorder. **(b)** Functional scores of phosphosites in "All" versus "Phoslink", with the Wilcoxon rank-sum test p-value indicated above. "All" represents all phosphosites analyzed, and "Phoslink"refers to those identified by our method as significantly regulating downstream protein expression. The left panel shows the scores from Supplementary Table 3 of the referenced paper, and the right panel presents scores recalculated using funscoR. **(c)** Functional scores of phospho-regulators.

Table S1. Full list of 109 phospho-regulators.

Gene Name	Residue	Position
ADD1	S	600
AEBP1	S	347
AHNAK2	S	5714
AKAP12	S	598
AKAP12	S	696
AKAP13	S	2345
ANK3	S	959
ARHGAP32	S	871
ARHGEF11	S	1457
ARHGEF11	T	590
BIN2	S	357
CDC42BPA	S	1719

CDK13	S	439
CERS4	S	342
CERS4	S	347
CROCC	S	512
DNMT1	S	143
DOCK6	S	176
DOCK7	S	1383
EEPD1	S	173
ELK3	S	396
EPB41L3	S	88
ESF1	S	663
ESYT2	S	739
FAM83E	S	351
FKBP15	S	960
GIT2	S	415
GORASP2	S	451
HERC1	S	1512
IGF2R	S	2479
JUP	S	182
KRI1	S	136
LMO7	S	1026
LMO7	S	246
LSP1	S	252
LSP1	T	175
LTBP2	S	1395
MACF1	S	4836
MACF1	S	5808
MAP4	S	941
MAP7D1	S	112
MAP7D1	S	552
MAP7D1	S	70
MLPH	T	458
MSH6	S	261
MSH6	S	830
MYEF2	S	17
MYLK	S	1438
MYLK	S	305
NADK	S	46
NBEAL2	S	2739
NES	S	931
NKTR	S	1146
NOC2L	S	672
NOC2L	S	673
NOL9	S	4
NUMA1	S	1760
NUP153	S	516
PARP4	T	101
PCNT	S	2355

PGM2	S	165
PHLDB2	S	513
PLEKHN1	S	559
PML	T	196
PPIG	S	397
PTPN12	S	332
PTPN12	S	673
RAP1GAP	S	484
RETREG2	S	385
RREB1	S	161
RRP1B	S	513
SDC2	S	187
SHC1	S	139
SLC2A9	S	9
SON	S	2013
SP110	S	256
SP110	S	380
SP3	S	73
SRRM2	S	1083
SRRM2	S	901
SVIL	S	261
SVIL	S	270
SYNE2	S	4108
SYNM	S	1181
SYNPO2	S	274
TACC2	S	2072
TASOR2	S	1025
TBC1D5	S	539
TJP2	S	292
TJP2	S	294
TJP2	S	296
TMEM176B	S	245
TNKS1BP1	S	712
TNS1	S	1119
TNS1	S	1307
TNS1	S	783
TNS1	S	907
TNS1	T	379
TNS2	S	830
UBE3B	S	419
VCAN	S	1351
VCAN	S	2112
VSIR	S	235
WDR75	S	779
WDR75	S	782
WNK1	S	174
YEATS2	S	465
ZC3H13	S	877

ZC3HAV1	S	275
